# An Evidenced-Based Review of Emergency Target Blood Pressure Management for Acute Aortic Dissection

**DOI:** 10.1155/2023/8392732

**Published:** 2023-07-20

**Authors:** Hankui Hu, Zhoupeng Wu

**Affiliations:** Department of Vascular Surgery, West China Hospital, 37 Guoxue Alley, Chengdu 610041, Sichuan, China

## Abstract

**Objective:**

To summarize the best evidence of emergency target blood pressure management for acute aortic dissection and provide guidance for evidence-based practice of emergency target blood pressure management.

**Methods:**

According to the “6S” evidence pyramid model, the evidence of emergency target blood pressure management of acute aortic dissection in various foreign databases and websites of professional associations from January 1, 2010, to August 1, 2022, was retrieved, including clinical decision-making, guidelines, expert consensus, systematic reviews, randomized controlled trials, cohort studies, and case series. Two researchers used the corresponding document quality evaluation tools to evaluate the documents and extracted and summarized the evidence of documents above grade B.

**Results:**

A total of 17 articles were included, including 6 clinical decision-making articles, 5 guidelines, 2 expert consensus articles, 1 systematic review article, 1 randomized controlled trial article, 1 cohort study article, and 1 case series article, forming 36 best evidences, including 9 topics, which are target value setting, management strategy, disease observation, medical history collection, monitoring methods, vasoactive drugs, nonvasoactive drugs, related examinations, and patient education.

**Conclusion:**

The best evidence summarized provides a reference for doctors and nurses in the emergency department to manage the emergency target blood pressure of patients with acute aortic dissection. It is recommended that doctors and nurses in the emergency department follow the best evidence summarized to develop individualized target blood pressure management plan for patients.

## 1. Introduction

Acute aortic dissection (AAD) refers to a life-threatening aortic disease [1]. Penetrating aortic ulcers, aortic intramural hematoma, and periaortic hematoma are variants of typical aortic dissection [2]. At present, the annual incidence rate of AAD is about 2.8/100000 [3]. About 1/3 of the untreated AAD patients died within the first 24 hours of the onset, and the mortality rate was more than half within 48 hours [4]. Some studies have shown that the incidence rate of AAD with hypertension is 50.1%∼75.9% [5]. Uncontrolled hypertension not only promotes the progress of dissection but also damages target organs. It is the most important and intervenable risk factor for AAD. Blood pressure management has become the initial treatment for AAD and has been throughout [4]. Some studies show that the 5-year survival rate of AAD patients with poor blood pressure management is about 60%, and that of those with good blood pressure management is as high as 95% [5]. At present, emergency blood pressure management is mostly based on personal clinical experience, and some medical staff are not even aware of the relevant knowledge of AAD blood pressure management and have not developed an individualized target blood pressure management plan for patients based on evidence-based evidence. The blood pressure management effect of AAD patients has not been significantly improved in the past 20 years [5]. In addition, according to the recommendations in the guide, some emergency department doctors are conservative in their medication. The above factors lead to a low rate of reaching the target blood pressure in emergency treatment and poor effects of blood pressure management [5]. Through a literature review, it was found that the existing evidence on AAD emergency target blood pressure management is scattered. Therefore, it is necessary to integrate the best evidence of AAD emergency target blood pressure management to provide reliable evidence-based medical evidence support for emergency clinical practice.

## 2. Methods

### 2.1. Establish the Question

According to the PIPOST [6] tool, the evidence-based question of this study is constructed, which is, “how to manage the target blood pressure of AAD patients.” P (population): patients diagnosed with acute aortic dissection; I (intervention): a series of intervention measures for target blood pressure management; P (professional): medical staff of the emergency department; O (outcome): target blood pressure reaching rate, hospital mortality; S (setting): emergency department of the hospital; and T (type of evidence): relevant guidelines, randomized controlled trials, systematic reviews, and so on.

### 2.2. Retrieval Strategy

According to the “6S” evidence resource pyramid model [7], the computer searches with the English keywords of “aortic dissection/aortic aneurysm/aortic disease/aortic syndrome/dissection aneurysm/blood vessel dissection/intramural haematoma/penetrating atherosclerotic ulcer” and “hypertension/blood pressure management/hypotension/blood pressure control/homeodynamics” in UpToDate, Guidelines International Network (GIN), Scottish Intercollegiate Guidelines Network (SIGN), National Institute for Health and Care Excellence (NICE), Canadian Medical Clinical Practice Guidelines (CPG), WHO, Maimaitong Registered Nurses' Association of Ontario (RNAO), American Heart Association (AHA), European Society of Cardiology (ESC), JBI, The Cochrane Library, PubMed, Embase, CINAHL, SinoMed, Web of science, and Clinical trials are retrieved from January 1, 2010, to August 1, 2022.

### 2.3. Inclusion and Exclusion Criteria of Literature

Inclusion criteria were as follows: The patients were nontraumatic AAD patients aged ≥18 years; the research content was AAD blood pressure management; the types of evidence resources included clinical decision-making, guidelines, expert consensus, systematic reviews, randomized controlled trials, cohort studies, and case series. Exclusion criteria were as follows: the patients were pregnant; the quality of literature was below grade B; articles were published repeatedly or were unable to obtain the full text from; abstracts of the meeting; interpretation or summary of the guide; and updated old guide or expert consensus.

### 2.4. Literature Quality Evaluation Method

This study was independently evaluated by two doctors trained in evidence-based medicine. When the evaluation opinions of the two doctors were inconsistent, the decision was made by consulting evidence-based medicine experts. When the conclusions of evidence from different sources conflict, this study follows the inclusion principle of high-quality evidence first, the latest published authoritative literature second, and then the evidence-based evidence [8].

UpToDate belongs to the top resource of the evidence resource pyramid model, and the evidence conforming to the clinical situation is directly used. The guidelines adopt the AGREE II [9] (Appraisal of Guidelines for Research and Evaluation Instrument) evaluation tool. AMSTAR [10] evaluation tool is used for system reviews. Randomised controlled trials, cohort studies, and case series were evaluated using the corresponding evaluation tools of the Australian JBI Evidence-based Health Care Center Evaluation Standard (2016) [7].

### 2.5. Evidence Grade and Recommended Strength

**Figure 1:**
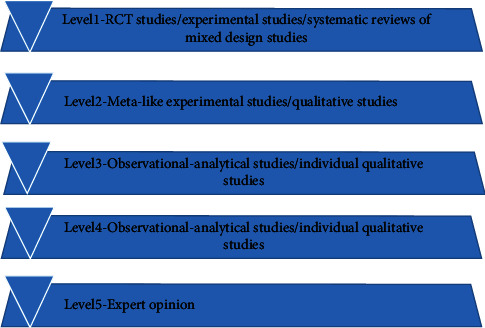
The evidence preclassification.

**Figure 2:**
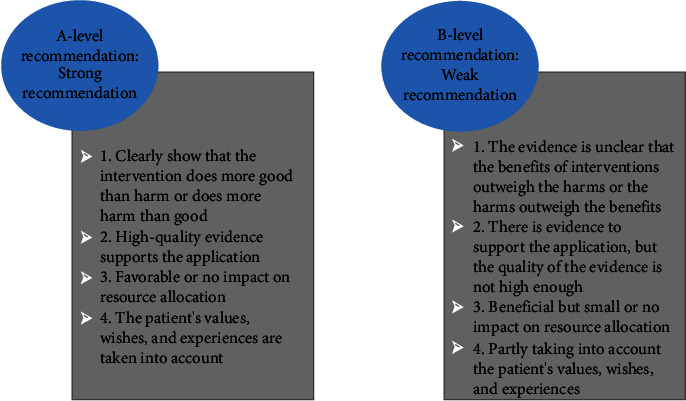
The evidence recommendation level system.

The evidence preclassification and evidence recommendation level system (2014) [8] of Australia's JBI Evidence-based Health Care Center was used to determine the evidence level and recommendation strength. According to the type of study, the level of evidence is divided into level 1∼5, and a correspondence expert group (all with evidence-based medicine/nursing background, including 2 emergency physicians, 4 nurses, 1 cardiac surgeon, and 1 vascular surgeon) is established. The recommendation strength of the evidence is determined according to the FAME attribute of the evidence (feasibility, appropriateness, clinical significance, effectiveness), including level A recommendation (strong recommendation) and level B recommendation (weak recommendation) (Figures 1 and 2).

## 3. Results

### 3.1. Literature Retrieval

**Figure 3:**
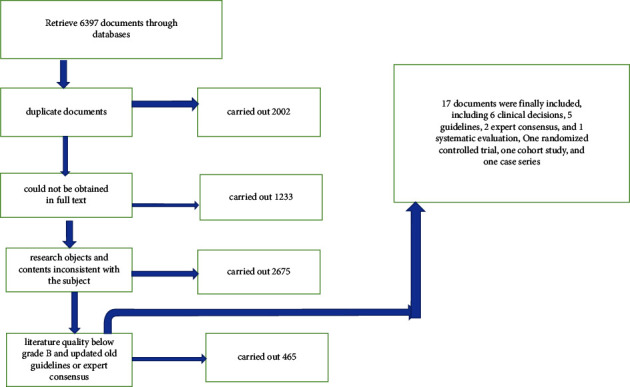
Literature search process.

A preliminary search of 6397 relevant documents, including 2002 duplicate documents, 1233 documents that could not be obtained in full text, 2675 documents with research objects and contents inconsistent with the subject, 465 documents with literature quality below grade B and updated old guidelines or expert consensus, was carried out, and 17 documents were finally included, which are 6 clinical decisions [2, 11–15], 5 guidelines [16–20], 2 expert consensus [21, 22], 1 systematic review [23], 1 randomized controlled trial [24], one cohort study [25], and one case series [26] (Figure 3 and Table 1).

### 3.2. Literature Quality Evaluation

**Figure 4:**
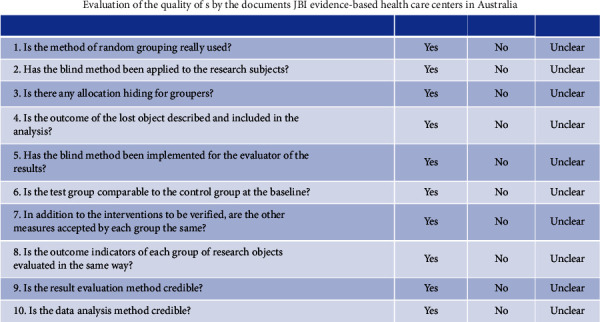
Evaluation of the quality of the documents in the ten items.

(1) A total of five guidelines were included, including the percentage of standardization in each field and the quality evaluation of the guidelines (Table 2). (2) Quality evaluation of expert consensus. A total of 2 expert consensus articles were included; all of the items were “Yes.” (3) Others include 1 systematic review, and all items are “Yes”; 1 randomized controlled trial, item 9 is “no,” 1 cohort study, item 3 is “unclear,” 1 case series, item 7 is “unclear,” and the other items are “yes.”) (The items in Figure 4) (Table 3)

### 3.3. Summary of Evidence

Through the extraction and integration of evidence, we finally summarized the evidence from 9 aspects, including target blood pressure setting, management strategy, disease observation, medical history collection, monitoring means, vasoactive drugs, nonvasoactive drugs, relevant examinations, and patient education, and formed 36 best evidence (Table 4).

## 4. Discussion

This study analyzed and recommended evidence from the following three aspects: target blood pressure setting and management strategies; vascular active drugs and other relative drugs; and patient education.

Evidence from this study (1–8) indicated that the doctors in the emergency department need to develop initial target blood pressure values, time to reach target values, and target blood pressure management strategies based on individual differences in patients. The initial treatment of AAD is to quickly stabilize the patient's hemodynamics under tolerable conditions [2, 27] and slow down the progression of the disease by reducing blood pressure and heart rate. Hypertension will lead to the rupture of the dissection, while Hypotension will affect the blood supply of important organs, so the systolic pressure should be kept within the ideal range of 100∼120 millimetre of mercury [2]. There is evidence to suggest that blood pressure <120/80 mmHg and heart rate <60/min during conservative treatment are beneficial for patient prognosis [11]. For patients with AAD rupture and hemodynamic instability, the systolic blood pressure level (80∼100 mmHg) is maintained at a low level, that is, the allowable hypotension [15], while ensuring adequate organ perfusion. Unlike hypertension management strategies that combine other diseases, AAD patients need to quickly (20–30 minutes) reduce blood pressure to the safe target range while ensuring sufficient organ perfusion [27, 28]. In addition, when making clinical decisions, it is also necessary to consider factors such as the patient's and their family members' attitudes towards treatment and expectations for prognosis. By establishing an efficient and cooperative multidisciplinary diagnosis and treatment team (including an emergency medical team, cardiac surgery, vascular surgery, and imaging department), the emergency quality of AAD patients can be improved [29].

The evidence of this study (9–19) emphasized that AAD patients should receive monitoring and treatment in the emergency department. Emergency physicians closely observe to avoid potential factors that affect blood pressure changes and effectively reduce related complications and mortality [30]. Studies have found that poor visceral perfusion is an important complication of AAD [31]. For example, low back pain with a sudden decrease in urine output or an increase in creatinine often involves renal artery dissection. Hypotension often indicates cardiac tamponade, abdominal pain, and an increase in lactic acid with or without melena are manifestations involving Mesentery arteries, which should be given sufficient attention by emergency physicians. The high-risk signs of AAD are pulse pressure difference of both upper limbs >20 mmHg [32], hypotension, asymmetric pulse intensity of both upper limbs, or disappearance of one side [16]. Emergency doctors can also evaluate the progress of the anatomy through dynamic observation. It is also important to guide the development of blood pressure management plans by collecting information about whether patients have a high-risk medical history and a special medication history. In terms of monitoring methods, it is necessary to measure the blood pressure of the patient's limbs. Invasive arterial blood pressure monitoring can quickly identify changes in patients' blood pressure [16], which has important clinical significance for observing the condition and adjusting medication in a timely manner. However, invasive procedures such as arterial puncture are required, which has certain technical requirements for emergency treatment. Although there is evidence that pulmonary artery floating catheter and central venous pressure monitoring can be used when patients have serious hemodynamic disorders, this requires a certain level of professional skills and preparation time and is not recommended as an emergency monitoring method [19].

The evidence in this study (20–31) elucidated the selection and considerations of drugs. In drug selection, *β* receptor blockers are the first choice for the treatment of AAD [32], except for contraindication such as asthma and atrioventricular block. If using *β* receptor blockers still cannot achieve the expected antihypertensive goals, it is recommended to [4, 25] use two or more antihypertensive drugs together, such as urapidil, sodium nitroprusside, and diltiazem, to achieve the goal of rapid hypotension. Emergency doctors need to evaluate whether there are contraindication when selecting drugs. The medication method is micropump intravenous maintenance. During the medication process, close attention should be paid to whether there are any adverse reactions. The choice of drugs for hypotension patients is very limited and must be used with caution. At the same time, actively search for the causes of hypotension and closely observe the changes in the condition [20]. The severe pain caused by AAD can lead to the release of endogenous neurotransmitters in the patient's body, leading to increased blood pressure and poor subjective experience. Therefore, morphine, pethidine, and other powerful analgesics should be given actively [21]. Some studies have shown that traditional Chinese medicine can also be used as an auxiliary drug for AAD blood pressure management, but it has not been widely used in clinical practice and is not recommended for emergency treatment [32].

The evidence from this study (32–34) clearly describes that emergency physicians should select the best diagnostic method based on the patient's hemodynamic status through careful and rapid comprehensive evaluation. This can not only quickly determine whether the target organs such as the kidney and intestine are damaged but also determine the location and extent of aortic dissection. In addition, there is evidence (35-36) that emergency physicians should provide patients with a comfortable diagnosis and treatment environment, necessary disease-related knowledge education, and psychological intervention, fully utilize a good social support system, alleviate negative emotions such as fear and anxiety, and avoid excessive blood pressure fluctuations. Meanwhile, smoking is the second largest risk factor for AAD, and strict smoking cessation interventions should be carried out for patients [31, 32]. Guide them to stay absolutely in bed and reduce behaviors that lead to chest and abdominal pressure and elevated blood pressure, such as forced coughing and defecation.

## 5. Limitations

Due to limitations or potential bias in individual studies, the assessment tools we used did not allow for such individual studies and could only target most of the literature. In addition, due to limitations of the review process, it could not address potential publication bias or the possibility of missing relevant studies.

## 6. Conclusion

This study summarizes the best evidence for target blood pressure management in an AAD emergency. This study provides the best strategy for blood pressure management. At the same time, the doctors and nurses in the emergency department should also consider the patient's situation and the family's expectations in an all-round way, formulate an individualized target blood pressure management plan, fully analyze the factors that promote and hinder the application of the evidence, and prudently apply the evidence to the clinic. In addition, emergency management personnel should actively improve and standardize blood pressure management rules and regulations, carry out training, and promote the transformation of knowledge and skills into practice.

## Figures and Tables

**Table 1:** Basic characteristics of documents.

Author	Literature sources	Type of literature	Title of literature	Publication/update	Citations
Warren	UpToDate	Clinical decision	Overview of acute aortic dissection and other acute aortic syndromes	2020	108
James and Warren	UpToDate	Clinical decision	Management of acute aortic dissection	2019	69
James and Warren	UpToDate	Clinical decision	Clinical features and diagnosis of acute aortic dissection	2021	153
Jeffrey and Robert	UpToDate	Clinical decision	Management of symptomatic (nonruptured) and ruptured abdominal aortic aneurysm	2021	70
William and Joseph	UpToDate	Clinical decision	Evaluation and treatment of hypertensive emergencies in adults	2021	25
William and Joseph	UpToDate	Clinical decision	Drugs used for the treatment of hypertensive emergencies	2019	16
Hiratzka	PubMed	Guideline	Guidelines for the diagnosis and management of patients with thoracic aortic disease	2010	827
Erbel	PubMed	Guideline	Guidelines on the diagnosis and treatment of aortic diseases: Document covering acute and chronic aortic diseases of the thoracic and abdominal aorta of the adult	2014	550
Riambau	PubMed	Guideline	Editor' choice management of descending thoracic aorta diseases: Clinical practice guidelines of the European society for vascular surgery (ESVS)	2017	304
Chaikof	PubMed	Guideline	The society for vascular surgery practice guidelines on the care of patients with an abdominal aortic aneurysm	2018	774
Ohle	PubMed	Guideline	Diagnosing acute aortic syndrome: a Canadian clinical practice guideline	2020	60
Fattori	PubMed	Expert consensus	Interdisciplinary expert consensus document on management of type B aortic dissection	2013	78
Boodhwani	PubMed	Expert consensus	Canadian cardiovascular society position statement on the management of thoracic aortic disease	2014	103
Mussa	PubMed	Systematic review	Acute aortic dissection and intramural hematoma: a Systematic review	2016	69
Zhang	PubMed	Randomised control trial	Efficacy of “Pinggan formula” in controlling acute type B aortic dissection perioperative blood pressure: a randomized controlled clinical trial	2019	12
Liao	PubMed	Cohort study	A retrospective observational study to assess prescription pattern in patients with type B aortic dissection and treatment outcome	2016	26
Krenz	PubMed	Case series	Evaluation of esmolol for heart rate control in patients with acute aortic dissection	2021	8

**Table 2:** Quality evaluation of five guides.

Literature	Standardized percentage of each field (%)						Overall rating 1 (points)	Overall rating 2 (points)	Number of ≥60% areas (pcs)	Number of ≤30% areas (pcs)	Recommended level
	Scope and purpose	Participant	Rigour of guideline designation	Clarity of guide designation	Applicability of the guidelines	Independence of guideline writing					
Hiratzka	94.45	61.12	81.25	94.45	81.25	95.84	6	6	6	0	A
Erbel	86.12	52.75	66.67	86.12	66.67	91.67	4	5	4	0	B
Riambau	100.00	47.22	54.17	100.00	54.17	95.84	5	4	4	0	B
Chaikof	100.00	38.89	91.67	100.00	91.67	91.67	5	6	5	0	B
Ohle	83.34	86.11	77.08	83.34	77.08	95.84	6	6	6	0	A

**Table 3:** The quality documents.

Author	Literature sources	Type of literature	Title of literature	The quality of the documents
Warren	UpToDate	Clinical decision	Overview of acute aortic dissection and other acute aortic syndromes	All items are yes
James and Warren	UpToDate	Clinical decision	Management of acute aortic dissection	All items are yes
James and Warren	UpToDate	Clinical decision	Clinical features and diagnosis of acute aortic dissection	All items are yes
Jeffrey and Robert	UpToDate	Clinical decision	Management of symptomatic (nonruptured) and ruptured abdominal aortic aneurysm	All items are yes
William and Joseph	UpToDate	Clinical decision	Evaluation and treatment of hypertensive emergencies in adults	All items are yes
William and Joseph	UpToDate	Clinical decision	Drugs used for the treatment of hypertensive emergencies	All items are yes
Hiratzka	PubMed	Guideline	Guidelines for the diagnosis and management of patients with thoracic aortic disease	All items are yes
Erbel	PubMed	Guideline	Guidelines on the diagnosis and treatment of aortic diseases: Document covering acute and chronic aortic diseases of the thoracic and abdominal aorta of the adult	All items are yes
Riambau	PubMed	Guideline	Editor' choice management of descending thoracic aorta diseases: Clinical practice guidelines of the European society for vascular surgery (ESVS)	All items are yes
Chaikof	PubMed	Guideline	The society for vascular surgery practice guidelines on the care of patients with an abdominal aortic aneurysm	All items are yes
Ohle	PubMed	Guideline	Diagnosing acute aortic syndrome: a Canadian clinical practice guideline	All items are yes
Fattori	PubMed	Expert consensus	Interdisciplinary expert consensus document on management of type B aortic dissection	All items are yes
Boodhwani	PubMed	Expert consensus	Canadian cardiovascular society position statement on the management of thoracic aortic disease	All items are yes
Mussa	PubMed	Systematic review	Acute aortic dissection and intramural hematoma: a Systematic review	All items are yes
Zhang	PubMed	Randomised control trial	Efficacy of “Pinggan formula” in controlling acute type B aortic dissection perioperative blood pressure: a randomized controlled clinical trial	Item 9 is “no
Liao	PubMed	Cohort study	A retrospective observational study to assess prescription pattern in patients with type B aortic dissection and treatment outcome	Item 3 is “unclear
Krenz	PubMed	Case series	Evaluation of esmolol for heart rate control in patients with acute aortic dissection	Item 7 is “unclear”

**Table 4:** Summary of evidence of AAD emergency target blood pressure management.

Subject	Evidence summary	Level of evidence	Recommended class
Target value	(1) Assess the presence or absence of target organ damage and use this to determine target blood pressure values and the time to achieve it	1	A
	(2) Initial target: Strict target heart rate <60/min or loose target heart rate <80/min, systolic blood pressure 100–120 mmHg or <130/80 mmHg (in combination with diabetes or chronic renal failure); for hemodynamically unstable AAD ruptures, maintain systolic blood pressure. 130/80 mmHg (in combination with diabetes mellitus or chronic renal failure); for hemodynamically unstable AAD ruptures, maintain systolic blood pressure	4	A
	(3) The recommendation is to reduce to the initial target value within 20–30 min while ensuring organ perfusion	5	B

Management strategy	(4) Initial treatment is blood pressure and heart rate control	5	B
	(5) The “hypotensive haemostasis” strategy of restrictive fluid resuscitation is recommended for the haemodynamically unstable	1	A
	(6) Emergency surgery recommended for AAD rupture and hemodynamic instability	1	B
	(7) Decision-making must be individualised, taking into account patients' coexisting conditions (e.g., stroke, renal failure, and diabetes), age and the expectations of patients and families	1	A
	(8) Recommended multidisciplinary team treatment	5	A

Disease observation	(9) Recommend dynamic assessment of patient symptoms and hemodynamic status in the emergency room	1	A
	(10) The extent and severity of AAD involvement can be inferred from arterial pulses and blood pressure values at different locations	1	A
	(11) If hypertension is poorly controlled with increased creatinine, decreased hourly urine output or back pain, suspect the presence of renal ischaemia	1	A
	(12) Severe chest, abdominal, low back or back pain with hypotension, consider AAD rupture	1	A
	(13) The presence of an odd pulse should be checked for hypotension to assess for pericardial tamponade; when systolic blood pressure is <90 mmHg or shock index >1 and pericardial effusion is present 1 pericardiocentesis is not recommended when systolic blood pressure is <90 mmHg or shock index >1 and pericardial fluid is present 1	1	A
	(14) Hypotension, systolic pressure difference between the arms >20 mmHg or absence of arterial pulsation in the proximal limb are high-risk signs and may guide the initial diagnosis	1	A

History-taking	(15) Any history of hypertension, diabetes, aortic intervention, aortic valve disease, Marfan syndrome or family history	1	A
	(16) History of specific drug use, like cocaine, methamphetamine	1	A

Monitoring instruments	(17) Recommended measurement of blood pressure in the extremities	1	A
	(18) Recommended invasive arterial blood pressure test	1	A
	(19) SwanGanz catheter and central venous pressure monitoring when severe hemodynamic disturbances are present	1	B
Vasoactive agents	(20) Combined antihypertensive drug therapy is recommended	1	A
	(21) 2 large diameter peripheral venous catheters are recommended to facilitate drug administration	1	A
	(22) The recommended initial treatment is an intravenous infusion of a beta-blocker, esmolol preferred, administered as a loading dose of 250−500 *μ*g/kg over 1 min, followed by at a rate of 25 to 50 *μ*g·kg^−1^·min^−1^; maximum dose 300 *μ*g·kg^−1^·min^−1^	1	A
	(23) Beta-blockers are contraindicated (e.g., asthma, heart failure, chronic obstructive pulmonary disease, or atrioventricular block) or used with caution (athletes, aortic angiotensin-converting enzyme inhibitors or angiotensin receptor antagonists). Change nondihydropyridine calcium channel blockers, such as diltiazem, verapamil	5	A
	(24) If beta-blockers fail to lower blood pressure sufficiently, a combination of sodium nitroprusside at an initial dose of 0.25–0.50 *μ*g·kg^−1^·min^−1^ and a maximum dose of 10 *μ*g·kg^−1^·min^−1^ is recommended(no more than 10 min); use away from light and monitor closely for blood pressure changes and signs of cyanide toxicity; use with caution in renal insufficiency	1	A
	(25) For AAD involving coronary arteries, nitroglycerin is recommended at a starting dose of 5 *μ*g/min and a maximum dose of 100 *μ*g/min, with observation for headache and tachycardia tachycardia	1	A
	(26) For patients with renal impairment, fenoldopam is recommended at a starting dose of 0.1 *μ*g·kg^−1^·min^−1^, which can be adjusted every 15 min up to 1.6 *μ*g·kg^−1^·min^−1^, with caution or contraindicated in patients with glaucoma; with caution in patients with sulphite sensitivity	1	A
	(27) ACEI or other vasodilators are recommended when the heart rate is <60 beats/min and systolic blood pressure remains above 120 mmHg, maintain adequate end-stage organ perfusion	1	B
	(28) Vasodilators alone should not be used until the heart rate is controlled	1	A
	(29) There are limited medications available to treat AAD with hypotension, and moderate rehydration is recommended.need to be assessed before rehydration, vasopressin may also be used, close monitoring for signs of entrapment progression is required	1	B

Nonvascular active drugs	(30) Recommend analgesic treatment such as morphine and pethidine	1	A

Relevant examinations	(31) Auscultation of the heart rhythm, presence of murmurs and additional heart sounds	1	A
	(32) Complete ECG, routine urine, routine blood, biochemistry, cardiac markers, coagulation, arterial blood gases, cephalothoracic and cephalothorax and abdomen CT or transesophageal echocardiography to assess for target organ damage	5	A
	(33) Aortic CT angiography is recommended for hemodynamically stable patients; emergency bedside transesophageal echocardiography or transthoracic wall echocardiography is recommended for unstable patients; magnetic resonance angiography is usually the test of choice for hemodynamically stable patients with renal insufficiency	3	A

Patient education	(34) Absolute bed rest and avoid strenuous activity, heavy lifting, coughing and bowel movements	1	A
	(35) Recommend suspension of cocaine, bupropion, varenicline, and so on	1	A
	(36) Inform patients and their families of the risk factors associated with hypertension, recommend smoking cessation, educate them about the disease and provide psychological support	1	A
